# Soil δ^13^C and δ^15^N baselines clarify biogeographic heterogeneity in isotopic discrimination of European badgers (*Meles meles*)

**DOI:** 10.1038/s41598-021-04011-2

**Published:** 2022-01-07

**Authors:** Shay T. Mullineaux, Berit Kostka, Luc Rock, Neil Ogle, Nikki J. Marks, Rory Doherty, Chris Harrod, W. Ian Montgomery, D. Michael Scantlebury

**Affiliations:** 1 School of Biological Sciences, Queen’s University Belfast, 1-33 Chlorine Gardens, Belfast, BT9 5AJ UK; 2 School of Natural and Built Environment, Queen’s University Belfast, David Keir Building, Stranmillis Road, Belfast, BT9 5AG UK; 3 Instituto de Ciencias Naturales Alexander Von Humboldt, Universidad de Antofagasta, Avenida Angamos 601, Antofagasta, Chile; 4 Universidad de Antofagasta Stable Isotope Facility (UASIF), Universidad de Antofagasta, Avenida Angamos 601, Antofagasta, Chile; 5 Shell Global Solutions International B.V., Amsterdam, The Netherlands

**Keywords:** Biological techniques, Ecology, Zoology, Biogeochemistry, Ecology, Environmental sciences

## Abstract

Isotopic techniques have been used to study phenomena in the geological, environmental, and ecological sciences. For example, isotopic values of multiple elements elucidate the pathways energy and nutrients take in the environment. Isoscapes interpolate isotopic values across a geographical surface and are used to study environmental processes in space and time. Thus, isoscapes can reveal ecological shifts at local scales, and show distribution thresholds in the wider environment at the macro-scale. This study demonstrates a further application of isoscapes, using soil isoscapes of ^13^C/^12^C and ^15^N/^14^N as an environmental baseline, to understand variation in trophic ecology across a population of Eurasian badgers (*Meles meles*) at a regional scale. The use of soil isoscapes reduced error, and elevated the statistical signal, where aggregated badger hairs were used, and where individuals were identified using genetic microarray analysis. Stable isotope values were affected by land-use type, elevation, and meteorology. Badgers in lowland habitats had diets richer in protein and were adversely affected by poor weather conditions in all land classes. It is concluded that soil isoscapes are an effective way of reducing confounding biases in macroscale, isotopic studies. The method elucidated variation in the trophic and spatial ecology of economically important taxa at a landscape level. These results have implications for the management of badgers and other carnivores with omnivorous tendencies in heterogeneous landscapes.

## Introduction

Stable isotopic analysis is a standard tool in the ecologist’s toolbox—it is routinely used to measure the transference of energy through biological systems and define sources of nutrition, across a range of molecules and tissue types, each with their own rates of assimilation and turnover for the ratios of ^13^C/^12^C and ^15^N/^14^N^1,2^. The movement of nutrients is measured through a food chain, with each transfer representing the movement of energy from source to sink within a food web. Carbon stable isotope values (δ^13^C) typically differentiates between C3, C4 and CAM photosynthesis, water-use efficiency, and stress, and terrestrial vs. aquatic environments^3,4^. Nitrogen identifies the levels of protein in an organism’s diet. Together, these ratios identify the energy sources fuelling a consumer and its trophic position^5–7^. Discrimination patterns and turnover rates in individual tissues/organisms vary temporally, and must be accounted for, to avoid misinterpreting noise from the environment^8,9^. This is difficult outside laboratory conditions, where environmental factors cannot be controlled. Consequently, the most effective approach for controlling for variation in isotopic routing, lipid content, biochemical pathways in tissue formation and diagenesis in fossil taxa, is the use of isotopic baselines. An isotopic baseline is defined as the isotopic ratio(s) before the trophic step of interest, the starting point of any isotopic study, and facilitates accurate interpretation of trophic patterns. In the absence of a baseline, intraspecies and interspecies comparisons can be made but only in special cases, e.g., fossil taxa in a shared rock deposit^10^. Baselines cannot be easily measured over macro geospatial scales using bulk stable isotopes. Isoscapes, GIS interpolated maps or process level models showing the variability of isotopic values over a landscape inferred from environmental samples offer an alternative solution in the absence of an isotopic baseline^11^.

Isoscapes permit an entire system to be visualised on a specific scale depending on sampling resolution and objective. At smaller scales, isoscapes have been used to describe biogeochemical and biophysical cycles and their interactions with microbial and plant communities^12^. At intermediary scales, isoscapes demonstrated patterns of nitrogen fixation in the invasive tree (*Acacia longifolia*), revealed interspecific competition with native species, and highlighted the effectiveness of the nitrogen fixing strategy in the invader^13^. On the macro-scale, δ^13^C and δ^18^O isoscapes were used to authenticate virgin olive oil from regions in Italy over a three-year period (2009–2011) using the isoscape framework proposed by 11^14^; and resolve patterns of endemism/ migration in animal species^15,16^. Bark and soil were used as environmental baselines for soil invertebrates, identifying niche partitions and ecological specialisations in different groups of organisms^17^. The potential to combine the isotopic and temporal complexity of an ecosystem, using GIS techniques for spatial display and analyses, opens further opportunities in diverse areas of environmental research and management^12^.

Isoscapes can shift over long time periods: δ^13^C, for example, decreases and increases as soil organic carbon is depleted or replenished, but this only occurs with respect to changes in land use over decades, for example δ^13^C can increase by 0.008–0.024‰ per year^18^. δ^15^N values of soils typically decrease with elevation and rise in mean temperature/precipitation but remain stable if climate and land use remain consistent^19,20^. δ^15^N ratios in soil are also affected by the nitrogen cycle, plant ecology, soil microbiota, type, and drainage, also mineral nitrogen is more readily retained in the soils of colder, wetter regions as less is lost through fractionation pathways^19,20^. In turn, temperate regions produce more persistent isoscapes than regions in the tropics, potentially making results and interpretations from temperate regions more consistent through time. Consequently, isoscapes in temperate regions could be a tool in ecological studies for an array of organisms, especially those with a fossorial ecology.

Studies of mammal trophic ecology using stable isotopes often employ complex mixing models to estimate the relative contribution of different putative foods to the assimilated diet of consumers. This technique requires reliable estimates of the isotopic shift (referred to as a trophic discrimination factor) between the food and the consumer tissue of interest. This is not always possible, but in certain conditions, comparisons may be made directly with the environment: for example, in palaeontology, by comparing a fossil with the surrounding rock^21−23^. Complex mixing models are frequently used in well studied systems, and are often the method of choice, but the application of multisource models to differentiate between food sources, is more difficult in omnivores or populations spread over a large geospatial scale^24^. Models using Bayesian methods coupled with Markov chain Monte Carlo, and stable *isotope* mixing models with complex hull or compositional transformations, can resolve dietary patterns in well documented populations and communities but still suffer when stable isotope values from food sources overlap, meaning that reliable source discrimination is impossible^25–29^. The influence of land-use, elevation, and the effects of other environmental parameters on consumer stable isotope values are also not always evident or resolvable. Alternative methods, utilising isoscapes to study intra-specific trends within a species, could offer more flexible tools for screening populations and their interactions with the environment, to allow for more direct understanding of widely distributed systems.

A potential study system combining stable isotope analysis and isoscapes is the European badger (*Meles meles*) and its interactions with soil at a landscape scale. This system is attractive due to badger ecology, which is well researched in the British Isles^30,31^: they live in relatively constant territorial units^32^, are omnivorous and consume a large range of food items^33,34^, many of which reside in or draw resources directly from soil. As fossorial animals (i.e., that partly live-in burrows dug from the soil), with permanent burrows or setts, soil plays for a critical role in the ecology of badgers^35^. Badger density varies with habitat and land class^36^ and shows considerable variation in behaviour both between clans, and between individuals within a clan^37,38^. Normally, isotopic studies of mammals require information on isotopic incorporation (tissue specific) which can vary temporally, and discrimination factors are either estimated from captive feeding studies, or more commonly, taken from related taxa in the literature^22^. However, the soil baseline is a constant, latent signal, within the data, and when used in a geospatial context may elucidate wider environmental trends and, thus, control for variation in land use, biogeochemical cycling etc. There is much published research in the palaeontological fields, comparing isotopic trends in animals with the environment, a technique which likely has much to offer in ecological investigations^23^.

The simplest model for estimating trophic position: (λ = (δnEconsumer − δnEbase)/Δn) where λ (trophic position), δnEconsumer (isotopic ratio of consumer), δnEbase (isotopic ratio of base) and Δn (enrichment factor per trophic level) is the basis of isotopic transference models^39^. This approach can estimate the trophic position of an organism given its δ^15^N value, that of the baseline and the trophic position of the baseline. However, food-webs are complex and estimating trophic position becomes increasingly difficult when multiple energy pathways (partitions) and diets (sinks) vary spatially, temporally and intraspecificly, widening ranges in isotopic ratio values, this is exacerbated by net zero contributions from food items and omnivorous diets^40^. Furthermore, an approach examining trends within a population could use the environment (soil) as alternative baseline and show intraspecific trends over macro geospatial scales.

The aim of the current study is to account for the geographic component of badger life history, by deducting the isotopic signal of the environmental (soil) baseline from the consumer (badger) (δ^n^I_consumer_ − δ^n^I_base_). This analysis will establish whether using soil as an environmental baseline, improves the isotopic trophic signal in the data, in a manner similar to food items in ecological studies. Consequently, this study investigates whether a soil isoscape baseline is useful in elucidating ecological variation in a badger population, and may help develop management strategies for bovine tuberculosis, transmissible by badgers, in heterogeneous landscapes comprising a mixture of optimal and suboptimal conditions. Bovine tuberculosis is an economically important factor in the agriculture of the UK and Ireland; annual costs approximately £100 M and £41 M respectively^41–43^. Current control methods draw on investigations that are limited to optimal habitats and land classes in lowlands, comprising a mix of improved pastures with deciduous woodland^44–47^. The effects of badger individuality, land use, and climate factors on isotopic analyses will be considered in comparison to the soil isotopic baseline.

**Figure 1:**
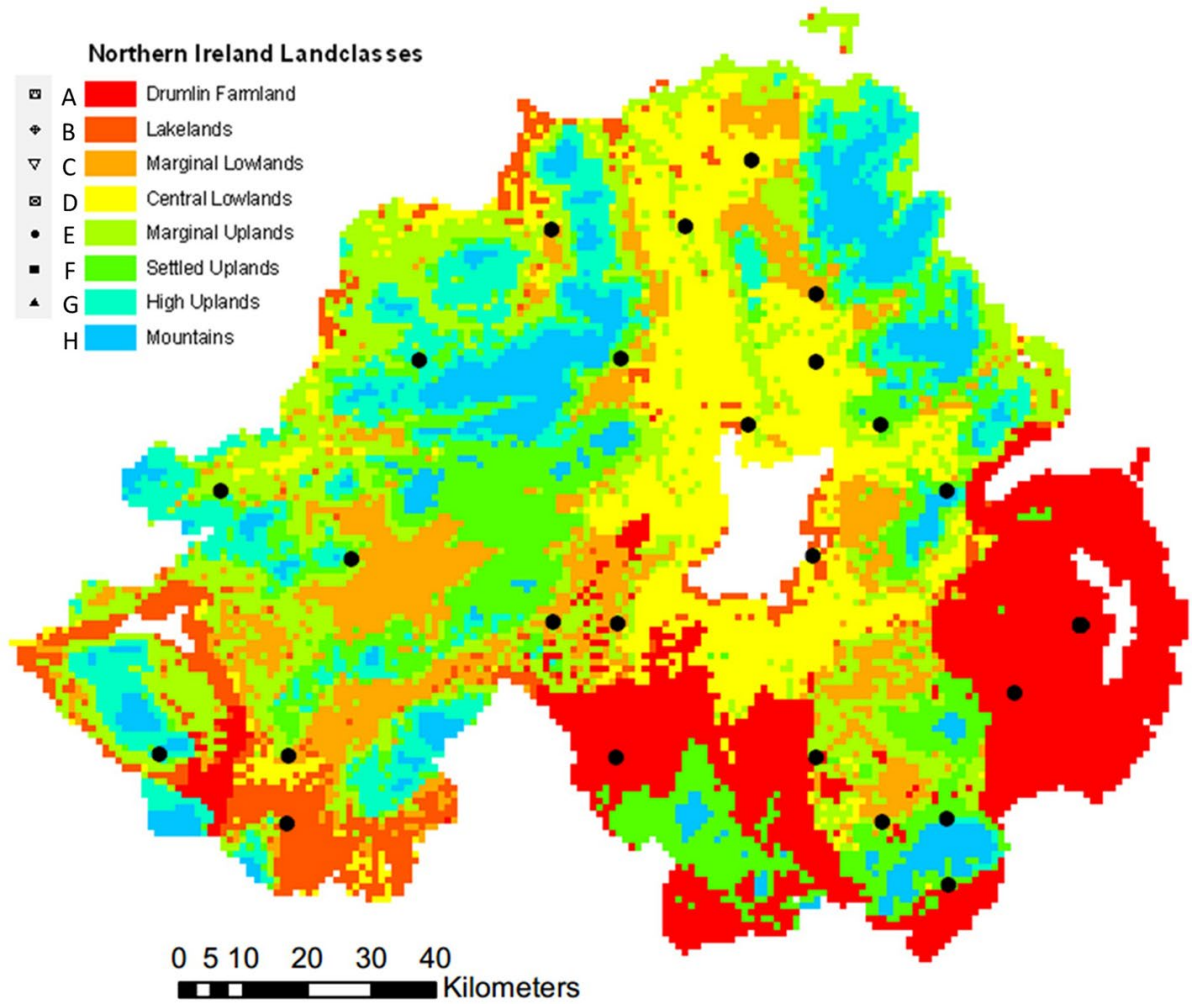
Land-use types across Northern Ireland, based on data in 49 and 50, categories (**A**–**G**) are included in this analysis, Mountains were excluded from analyses as this habitat is not commonly occupied by badgers (*Meles meles*) in Northern Ireland. Black dots denote sampled sett locations. This map was created in Arc GIS 10^48^.

This study examines the use of soil isoscapes as an environmental baseline for isotopic transference in an intraspecific comparison of European badgers (*Meles meles*) on a macro-scale across Northern Ireland (Fig. 1). This region is comprised of a high diversity in land-use and habitat types, which affect the availability of food items and the resultant isotopic fractions. The effects of these variables will be tested and the effect of the soil isoscape baseline evaluated.

## Methods

### Badger hair sampling and stable isotopic analysis

Badger hairs were collected between February and April 2009 from badger hair traps set outside setts throughout Northern Ireland and across a variety of land-use types (Fig. 1). Tufts of guard-hair were collected from free ranging badgers using non-invasive hair traps (consisting of lengths of barbed wire stretched between two upright poles near to sett entrances^49^). Hair traps were set seven days prior to sample collection. Subsequently, hair samples were collected every second day over a three-week period. Hairs were collected frequently to reduce sample degradation^50^. Hair samples were collected exclusively from rural locations to reduce chances of anthropogenic feeding. Work was undertaken under license from the UK Home Office. The identity of individual badgers was confirmed using microsatellite analysis of collected hairs^49^. These samples were then prepared for isotopic analysis by being cleaned in distilled water, dried for 48 h at 60 °C, cut, powdered by a pestle and mortar (weighing 0.5 mg ± 0.15 mg), and placed into tin capsules (6 × 4 mm, SerCon, Crewe, UK). The values of δ^13^C and δ^15^N were measured using isotope ratio continuous flow mass spectrometer (Delta V Advantage, Thermo Scientific) and the standard used was A-R041 L-Alanine (standard values, δ^13^C: − 23.3‰ and δ^15^N: − 5.6‰) and R041 (δ^15^N: − 5.56, δ^13^C: − 23.33), l-Glutamic Acid (δ^15^N: 47.60, δ^13^C: 37.63) and Leucine δ^15^N: 10.77, δ^13^C: − 30.52, were used periodically for calibration purposes, to counter drift and ensure a one-point (± 0.1‰) calibration for analytical precision of both isotopes. All analyses were conducted in the Chrono Centre, Queen’s University Belfast^49^.

### Soil sampling and isotopic analysis

Soil samples for isotopic analysis of C and N were obtained from the Geological Survey of Northern Ireland (GSNI). The 115 soil samples were collected as part of the Tellus geochemical mapping program of Northern Ireland and prepped in stages, firstly warmed at 30 °C to evaporate moisture, disaggregated with a ceramic pestle and mortar and 40 g subsample was milled (with a Retsch PM400 agate planetary ball mill) for 30–45 min until the sample was pulped until 95% of sample was < 32 µm and ready for chemical analysis^51^. Material was later processed at the Queen’s University Belfast Stable Isotope Facility^52^. The soil samples were prepared by a Thermo Delta V Plus Isotope Ratio Mass Spectrometer coupled to a Thermo Flash 1112 Elemental Analyser. Four standards were used during the analysis of the samples, including three international standards: benzoic acid (δ^13^C: − 28.8‰), potassium nitrate (δ^15^N: 4.7‰), Sucrose (δ^13^C: − 10.4‰) and one internal quality control: Leucine (δ^13^C: − 30.5, δ^15^N: 10.8‰). The soil samples provided the environmental baseline through which the isotopic fractionation within the hairs was calculated. Ordinary Kriging maps of δ^13^C and δ^15^N isotopic ratios throughout Northern Ireland were created from soil samples collected from the Tellus project and prepped for isotopic analysis (Supplementary Material 1). All maps were created in Arc GIS 10.5^48^.

### Genetic analysis

Samples of badger hair collected in traps potentially include hairs from more than one individual. Genetic microarray analysis was conducted to account for individual animals and avoid pseudo-replication (Supplementary Material 2). The genetic data were used to determine which hair samples belonged to a single individual, to ensure a single individual was used in calculation of mean isotopic data. This was conducted using 7 microsatellite loci and genotyping errors were controlled for with a conservative approach due to the inherent errors that occur when working with hair samples (Supplementary Material 2) (Genetic research was conducted at the NERC facility in Sheffield, UK). Where useful, individual-level identifications were used for some subsequent analyses (linear regression and K-means clustering) but not for GAM analysis as the information was averaged across different temporal periods.

### Statistical analysis

The original (Uncorrected) badger hair Isotopic ratios and deducted (Corrected) values (δnE_consumer_ − δnE_Isobase_), using the aggregated hair isotopic data and genetic microarray dataset taking individuals into account, were compared to assess the effect of the soil environmental isoscapes. Linear regression models were used to assess potential relationships between δ^13^C and δ^15^N and land classes. K-means clustering was used to analyse the isotopic ratios and site elevation to show which data set was best able to reproduce the original land classifications (Fig. 1) (Table S1, Supplementary Material 1)^53^.

UK Meteorological Office climate data from the period of February—April 2009 were taken from weather stations throughout NI and mean values from the nearest location to each sett was calculated for a period of fourteen days before hair sampling^54^. Five different variables daily mean temperature (°C), maximum temperature (°C), minimum temperature (°C), rainfall (mm) and total sunshine (hrs) were reduced via principal component analysis using the princomp function in R^55^. PC1 (Temperature) contained most of the variance for the three temperature variables and these were perpendicular to PC2 (Climate) rainfall (mm), whilst daily sunshine was distributed evenly between both. Both components were used in subsequent analysis (Supplementary Material 3)^56–58^. Due to the requirement of a time series, only the isotopic aggregated hair dataset was used, as the averaging across isotopic ratios required for the genetic dataset would cause spurious signals in the data. In the general additive models (GAMs), the response variable is graphed against predictor variable(s) which are altered by smooth functions to show non-linear trends in data. Factors including data on site elevation, date of sample collection, Temperature (PC1) and Climate (PC2) were used to assess subsequent changes in the colloquial ‘signal’ of the data to verify whether the deducted data were more effective at elucidating ecological trends given the abnormal snow event which occurred at this time^59^. All GAM analysis was conducted in the R package *mgcv*, k was set to the default value of − 1 and this was maintained for variables. All analysis was carried out in R 3.6.1^55^.

## Results

### Relationship between uncorrected and corrected (effect of soil baseline removed) δ^13^C and δ^15^N ratios

**Figure 2:**
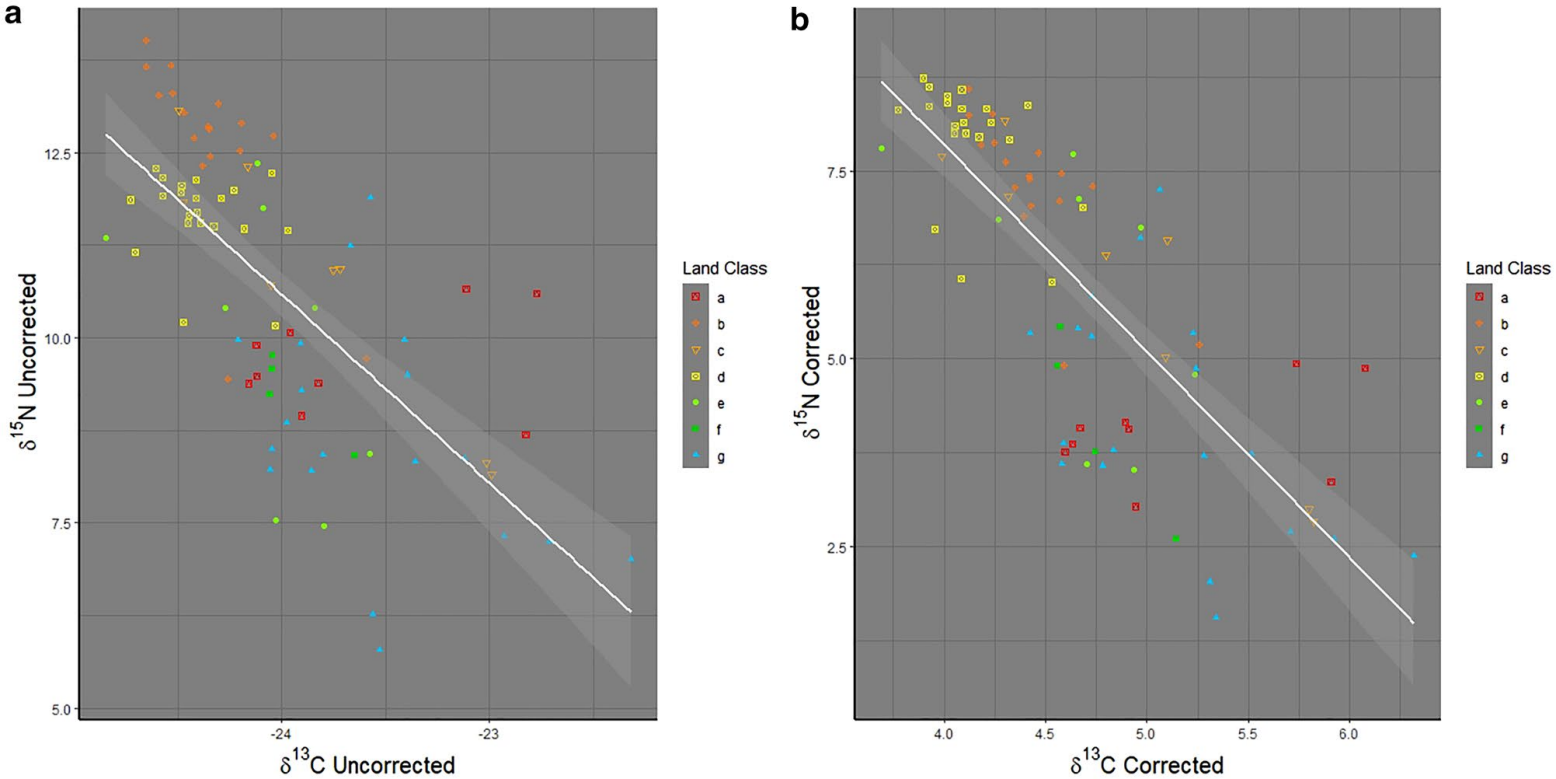
Linear Regression models of δ^13^C and δ^15^N for the hair dataset: (**a**) Uncorrected (**a**) (R^2^ = 0.477) and Corrected with the environmental baseline (**b**) (R^2^ = 0.599). Land classes: a (Drumlin Farmland), b (Lakelands), c (Marginal Lowlands), d (Central Lowlands), e (Marginal Uplands), f (Settled Uplands) and g (High Uplands).

**Figure 3:**
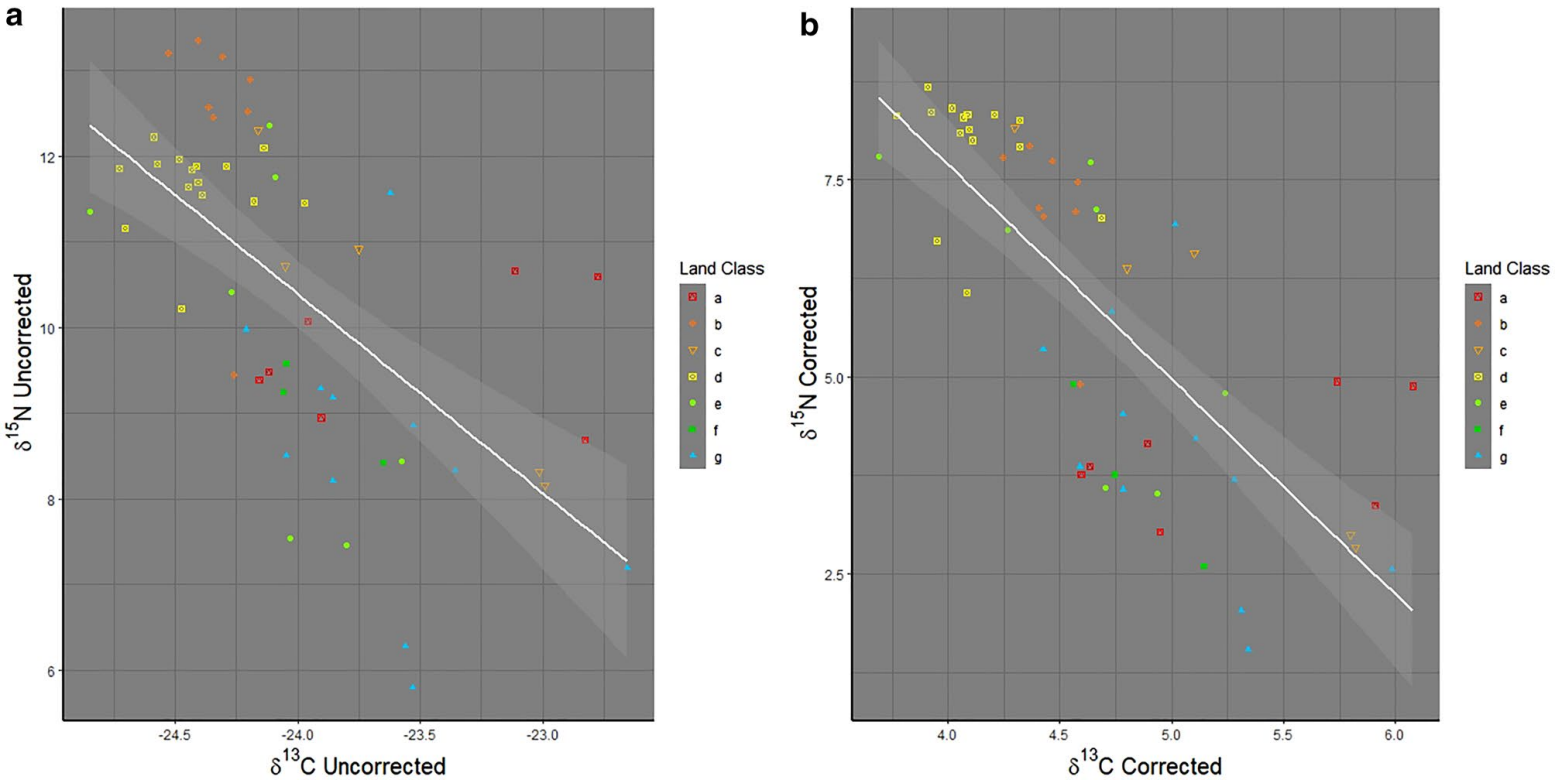
Linear Regression models of δ^13^C and δ^15^N for the genetic dataset corrected for individuals, Uncorrected (**a**) (R^2^ = 0.383) and Corrected with the environmental baseline (**b**) (R^2^ = 0.564). Land classes: a (Drumlin Farmland), b (Lakelands), c (Marginal Lowlands), d (Central Lowlands), e (Marginal Uplands), f (Settled Uplands) and g (High Uplands).

Linear regression fitted slopes for δ^13^C and δ^15^N values demonstrated a higher degree of covariance after using the soil isoscape as an environmental baseline (OLS R^2^: C uncorrected = 0.48, C corrected = 0.60, N uncorrected = 0.38, N corrected = 0.56) (Fig. 2a,b). This effect remained after reducing the dataset by substituting mean values for hairs from the same individuals (Fig. 3a,b). The land classes also retained a similar pattern of clustering around the regression line, though classes B and D near the top of the regression line become less distinct in the corrected dataset and classes A, E, F and G, remain on the lower half of the regression line but cluster more tightly in the corrected dataset (Figs. 2b, 3b). Class C, marginal lowlands, is the only land class type that is distributed along the length of the regression line. Badgers in land classes B and D, Lakelands, and Central Lowlands, had ^15^N enriched δ^15^N values, whilst those in land classes A, E, F and G, Drumlin Farmlands, Marginal Uplands, Settled Uplands, and High Uplands, were relatively low in ^15^N. Badgers present in land class C, Marginal Lowlands, show variability in their isotope ratios, indicating a higher degree of variability in foraging behaviour in this land class. Overall, data-points cluster more tightly in the corrected plots but still show distinct patterns.

### K-means clustering for site elevation, δ^13^C and δ^15^N ratios, resolving land-use patterns

**Figure 4:**
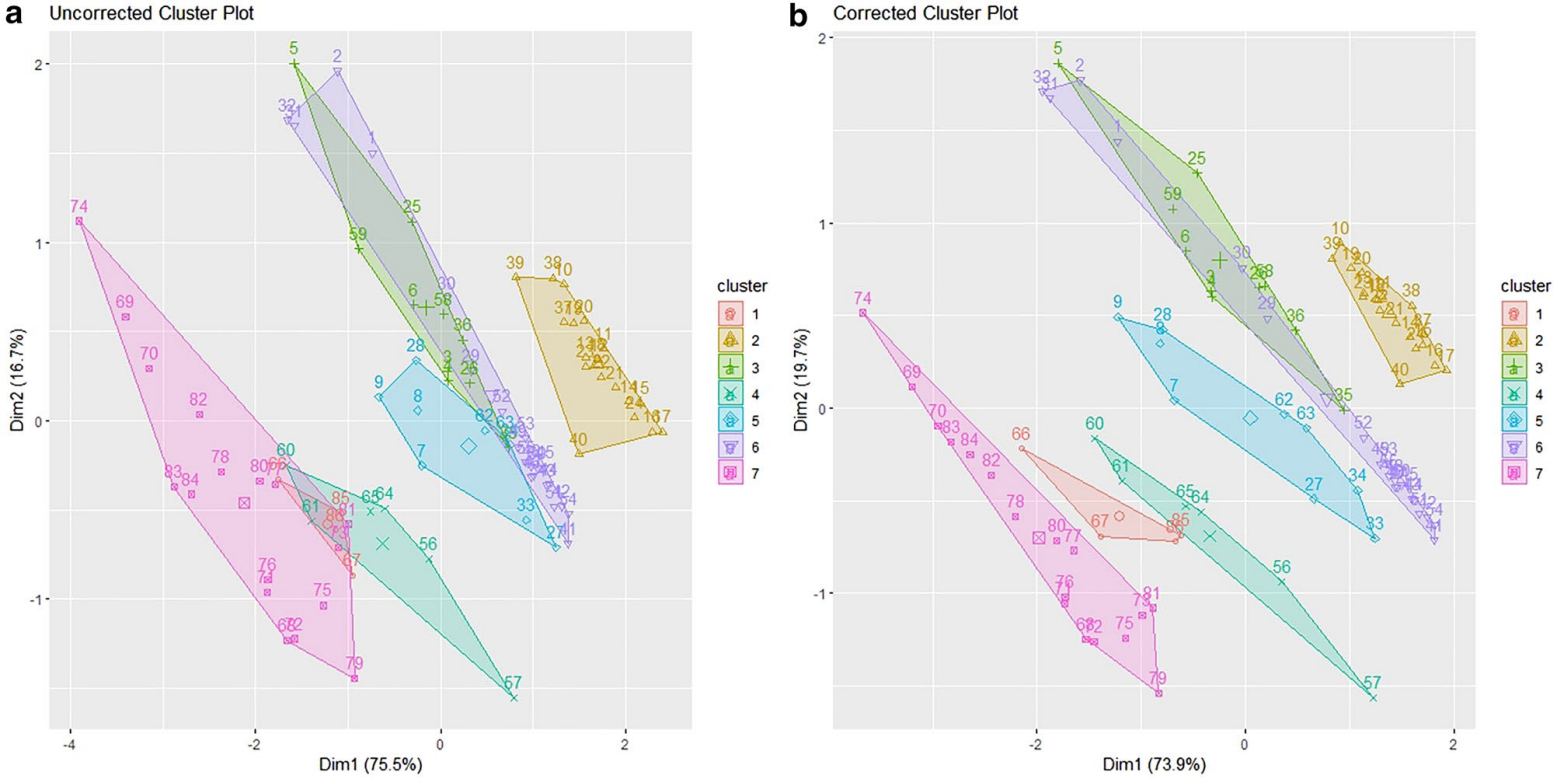
K-means cluster analysis of Site Elevation (m), δ^13^C and δ ^15^N for the hair dataset, Uncorrected K = 7 (**a**) and Corrected with the environmental baseline K = 7 (**b**). Dimension 1 loses 1.6% from A to B and Dimension 2 gains 3% of variance because of the baseline deduction.

**Figure 5:**
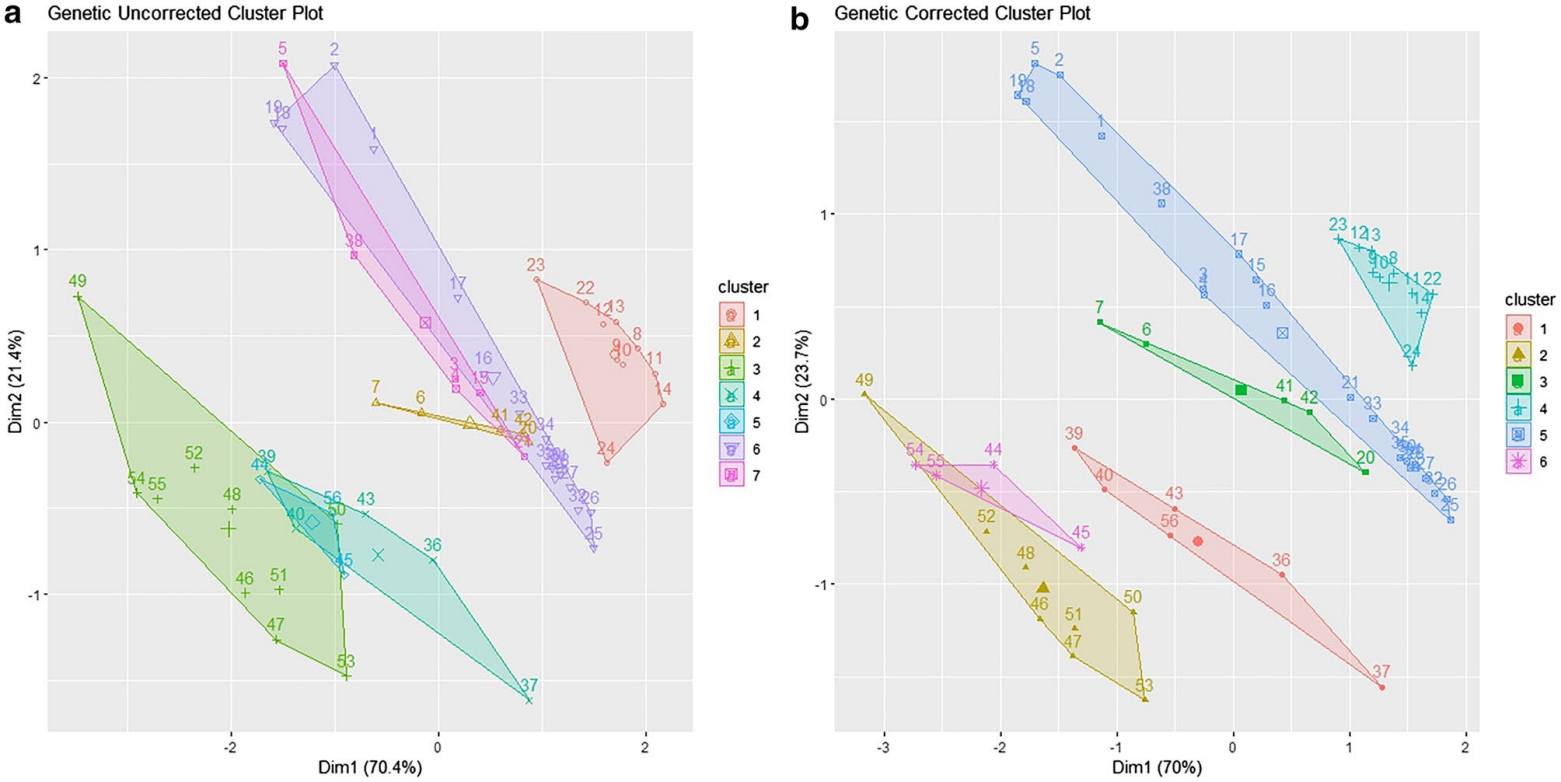
K-means cluster analysis of Site Elevation (m), δ^13^C and δ^15^N for the genetic dataset corrected for individuals, Uncorrected K = 7 (**a**) and Corrected with the environmental baseline K = 6 (**b**). Dimension 1 loses 0.4% from A to B and Dimension 2 gains 2.3% of variance because of the baseline deduction.

A K-means clustering analysis was conducted on site elevation (m), δ^13^C and δ^15^N ratios to determine whether land class type contributed to variability in the datasets. Both the hair dataset (Fig. 4) and the genetic-corrected dataset (Fig. 5) were analysed priorly with elbow plots and the *fviz_nbclust* function in the R package *factoextra*^60^ which identified K = 7 for uncorrected, corrected hair and uncorrected genetic datasets (Fig. 4a,b, Fig. 5a) and K = 6 for the genetic- corrected dataset (Fig. 5b). All figures show similar outcomes for the clustering of individual data points. The clusters for the uncorrected datasets (Figs. 4a, and 5a) are not as clearly resolved as the corrected values (Figs. 4b and 5b). Similar patterns are evident across all datasets with cluster 1 containing animals from Settled Uplands (F) and High Uplands (G); cluster 2: Lakelands (B), Marginal Lowlands (C) and Central Lowlands (D); cluster 3: Drumlin Farmlands (A), Lakelands (B), Marginal Lowlands (C) and Central Lowlands (D); cluster 4: Marginal Uplands (E) and Settled Uplands (F); cluster 5: Drumlin Farmlands (A), Marginal Lowlands (C) and Marginal Uplands (E); cluster 6: Drumlin Farmlands (A) and Central Lowlands (D); and cluster 7: Settled Uplands (F) and High Uplands (G). However, in Fig. 5a, clusters 6 and 7 swapped positions and this is likely due to the reduction of data points seen between the hair and genetic datasets. In Fig. 5b, after baseline deduction cluster 7 from Fig. 5a is absorbed into cluster 3, again showing the reduction in data points has affected the underlying signal in the data. Overall, the clusters are split between Lowland and Upland land classes, but site elevation is not the only factor contributing to the trend noted in the prior analysis. Overall, all groups became more distinct after baseline deduction and groups 3 and 6 remained affiliated in Fig. 4 but subsumed in Fig. 5.

### Relationship between temporal and environmental factors, and uncorrected and corrected δ^13^C and δ^15^N ratios

**Figure 6:**
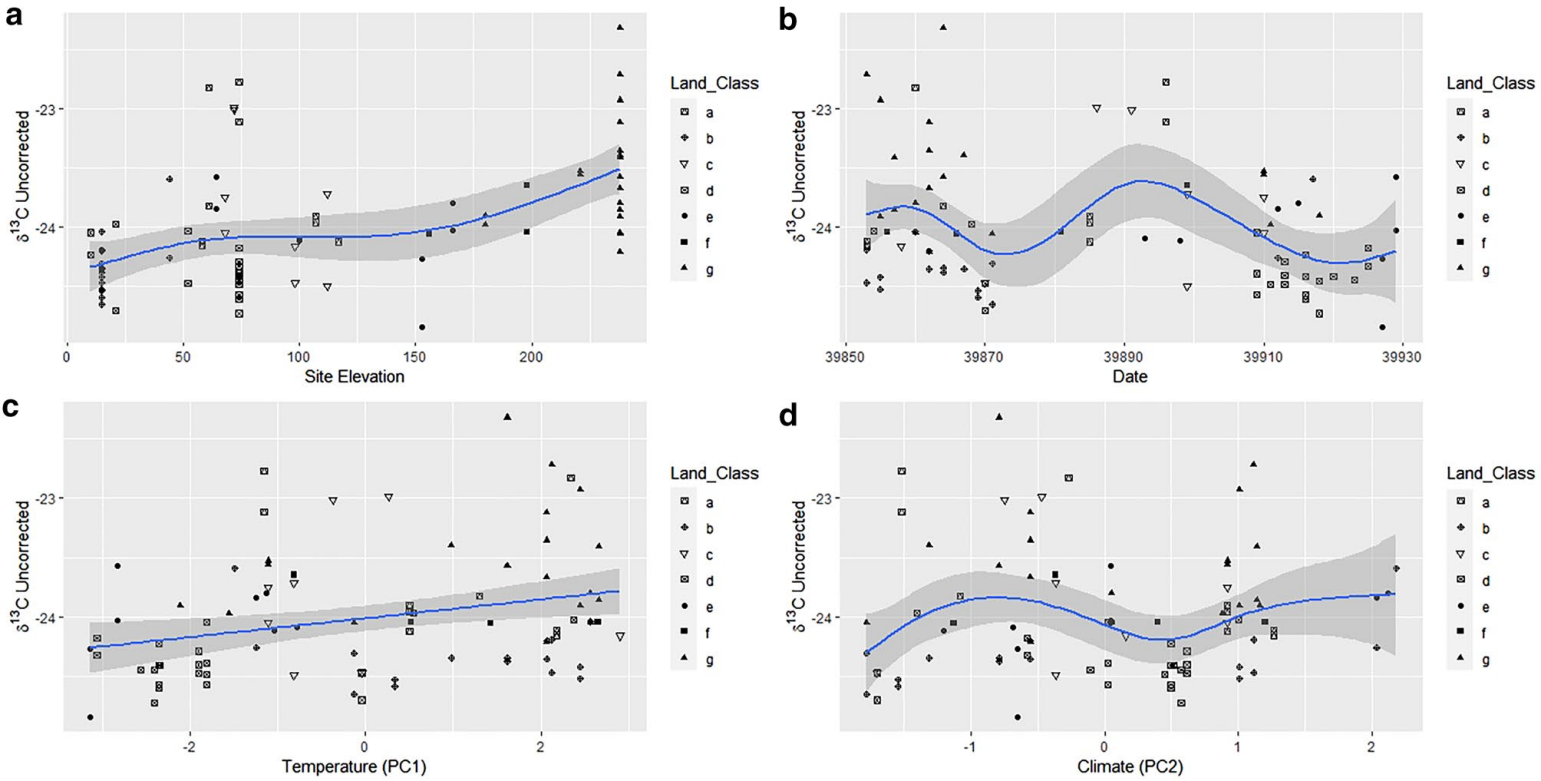
GAM factors smoothed for the hair dataset. Uncorrected δ^13^C versus (**a**) Site Elevation, (**b**) Date, (**c**) Temperature PC1, and (**d**) Climate PC2. Solid lines indicate the trend line, and the shaded area is the approximate 95% point-wise confidence interval.

**Figure 7:**
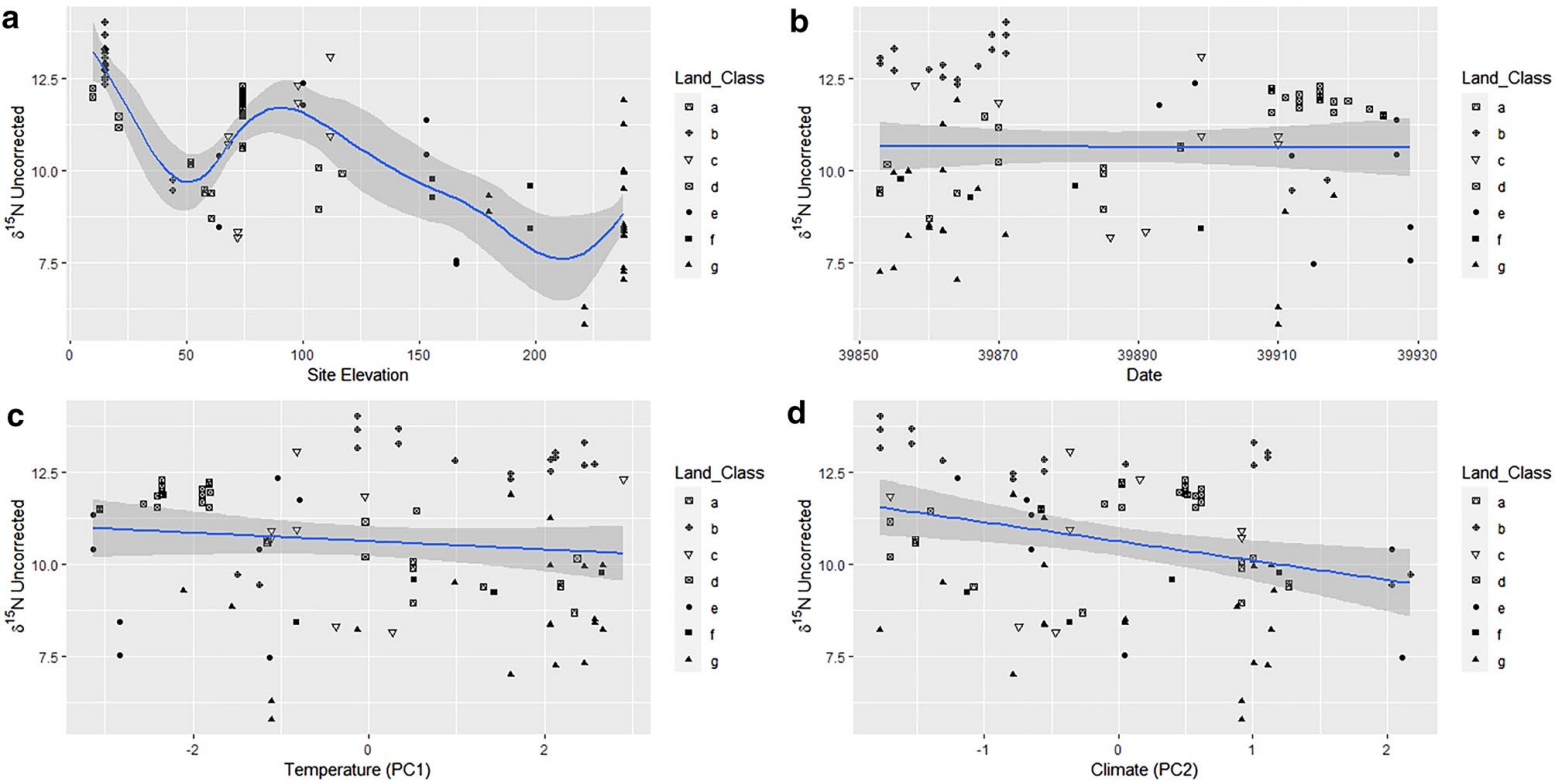
GAM factors smoothed for the hair dataset. Uncorrected δ^15^N versus (**a**) Site Elevation, (**b**) Date, (**c**) Temperature PC1, and (**d**) Climate PC2. Solid lines indicate the trend line, and the shaded area is the approximate 95% point-wise confidence interval.

**Figure 8:**
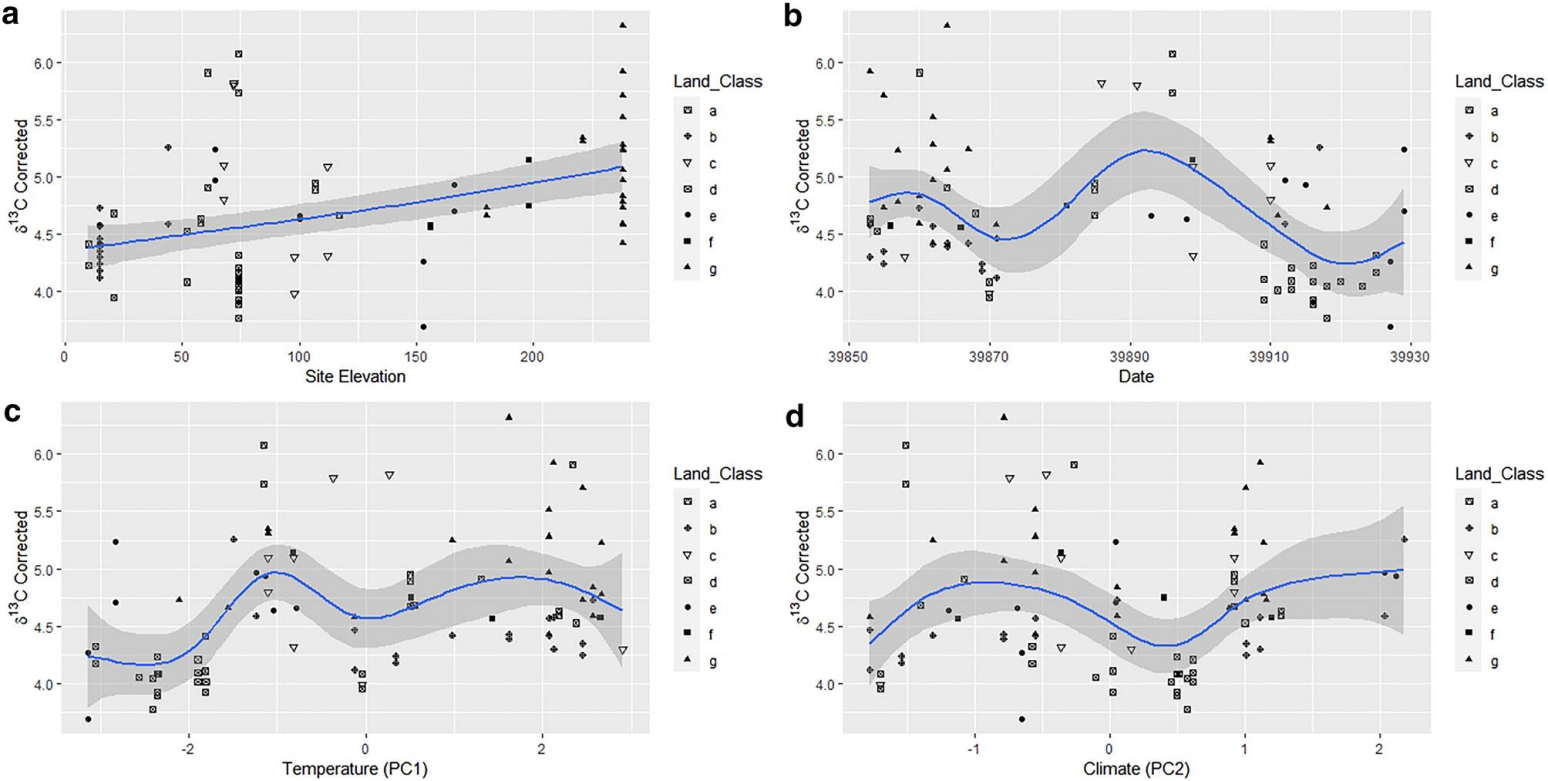
GAM factors smoothed for the hair dataset. Corrected δ^13^C versus (**a**) Site Elevation, (**b**) Date, (**c**) Temperature PC1 and, (**d**) Climate PC2. Solid lines indicate the trend line, and the shaded area is the approximate 95% point-wise confidence interval.

**Figure 9:**
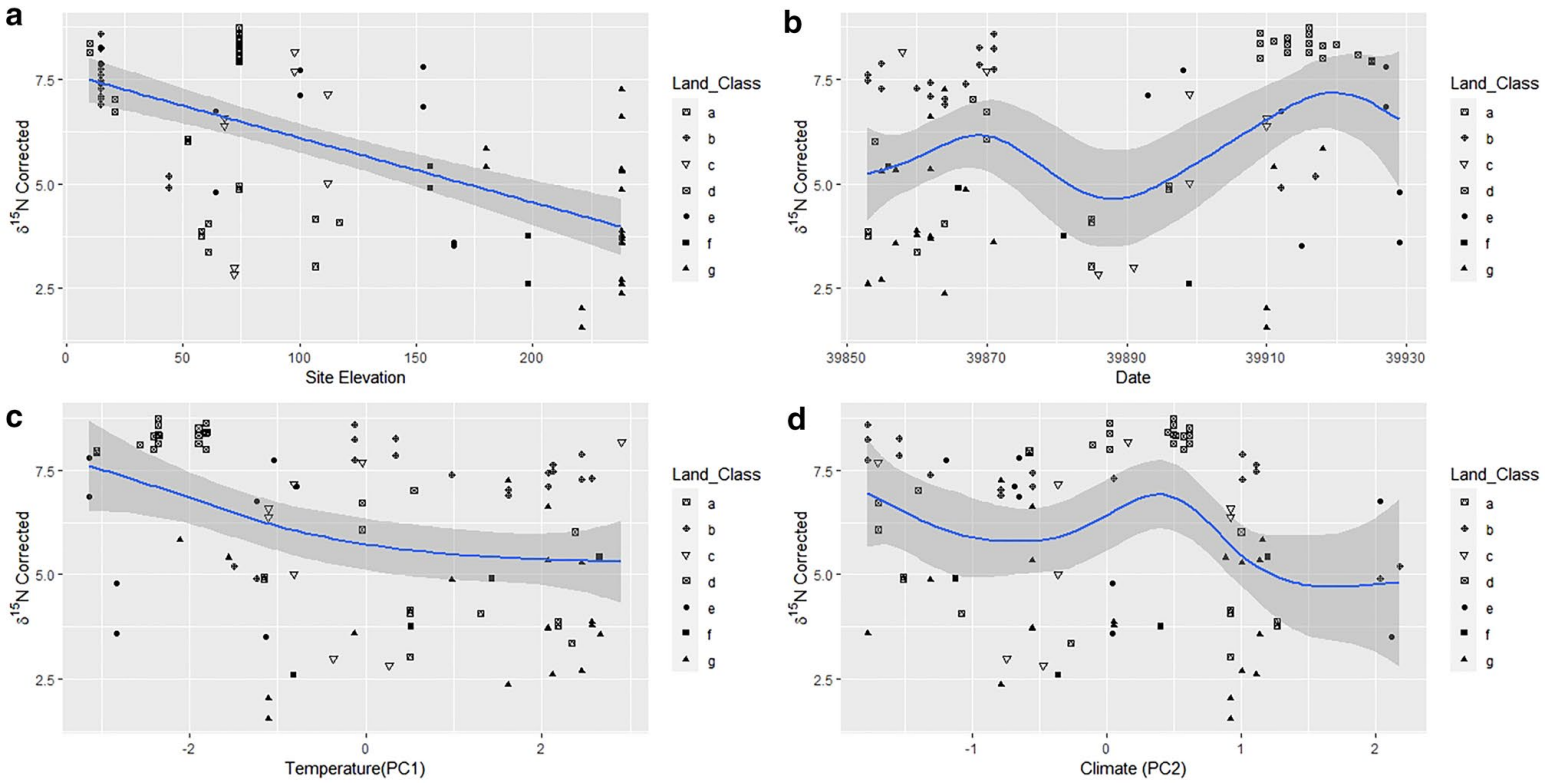
GAM factors smoothed for the hair dataset. Corrected δ^15^N versus (**a**) Site Elevation, (**b**) Date, (**c**) Temperature PC1, and (**d**) Climate PC2. Solid lines indicate the trend line, and the shaded area is the approximate 95% point-wise confidence interval.

GAM models were then run for uncorrected and corrected ratios and each of the factors: date, site elevation (m), temperature (PC1) and climate (PC2) were alternatively smoothed and correlated by uncorrected δ^13^C (Fig. 6), uncorrected δ^15^N (Fig. 7), corrected δ^13^C (Fig. 8), and corrected δ^15^N (Fig. 9) to assess what effect using soil as an environmental baseline had on the underlying signal in the data. When smoothed, all data types show a significant relationship with date and site elevation (m), all but uncorrected δ^15^N shows a significant relationship with temperature and only corrected δ^13^C correlates with climate (Table 1). The F-values of the smooths for the uncorrected data are mostly smaller than the corrected data. Only Corrected δ^13^C when paired with temperature or climate does not show this trend.

**Table 1:** F values and probability (p) of departure from no relationship and deviance explained values from GAM, following smoothing by date, site elevation (m), temperature (PC1) and climate (PC2); asterisks show levels of significance.

Isotopic treatment	Date		Site Elevation (m)		Temperature (PC1)		Climate (PC2)		Deviance explained value (%)
	F-value	p-value	F-value	p-value	F-value	p-value	F-value	p-value	
Uncorrected δ^13^C	4.62	< 0.001***	8.96	< 0.001 ***	2.92	0.0418 *	1.84	0.145	62.9
Uncorrected δ^15^N	2.77	0.0323 *	19.78	< 0.001***	1.47	0.221	0.000	0.988	78.2
Corrected δ^13^C	7.50	< 0.001***	8.18	< 0.001***	4.70	0.0038 **	2.14	0.096	69.5
Corrected δ^15^N	6.51	< 0.001***	14.00	< 0.001***	3.09	0.0311 *	0.03	0.862	79.2

Only corrected δ^15^N had a significant temporal relationship with date when not smoothed, whilst corrected δ^13^C had a similar but non-significant correlation. All datasets were significantly related with site elevation (m) and temperature, but only corrected δ^13^C correlated with climate (Table 2). The F-values indicate the same phenomenon documented above and again Corrected δ^13^C correlated with Temperature or Climate (Table 2).

**Table 2:** F values and probability (p) of departure from no relationship from GAM correlating by date, site elevation (m), temperature (PC1) and climate (PC2); asterisks show levels of significance.

Isotopic treatment	Date		Site elevation (m)		Temperature (PC1)		Climate (PC2)	
	F-value	p-value	F-value	p-value	F-value	p-value	F-value	p-value
Uncorrected δ^13^C	2.41	0.12	29.87	< 0.001***	4.42	0.037 *	2.5	0.12
Uncorrected δ^15^N	1.23	0.27	83.76	< 0.001***	5.72	0.019 *	1.48	0.23
Corrected δ^13^C	3.75	0.056	21.12	< 0.001***	6.26	0.014 *	2.84	0.096
Corrected δ^15^N	4.22	0.0437*	52.15	< 0.001***	5.61	0.02 *	0.043	0.84

The corrected datasets, where the soil baseline was deducted from the isotopic values of the hairs, showed a higher degree of curvature than their uncorrected equivalents. This was particularly evident between corrected and uncorrected δ^15^N datasets (Figs. 7 and 9). Conversely, site elevation (m) and date had similar trends in both datasets for δ^13^C (Figs. 6a,b, 8a,b). The relationships between isotopic ratios and temperature, PC1, (Figs. 6c, 8c) and climate, (PC2), (Figs. 6d, 8d) against δ^13^C show a marginal increase in the degree of plasticity in the data. In the relationships with the uncorrected δ^15^N dataset, only site elevation (m) (Fig. 7a) was curved, the other factors produced no relationship (Fig. 7b–d). The corrected δ^15^N trends were noticeably different, the curve for site elevation (m) (Fig. 9a) became more exaggerated and the trends for date, temperature, and climate (Fig. 9b–d) transitioned from horizontal lines to curves after deduction from the environmental baseline, indicating that the signal within the data has become more detectable. Additionally, the trends for corrected δ^13^C and δ^15^N, with date (Figs. 8b, 9b), temperature (Figs. 8c, 9c) and climate (Figs. 8d, 9d) show an inverse relationship. Thus, with the seasonal transition from February to April, the relationship between δ^13^C to δ^15^N in badger hair shifted in response to meteorological shifts and δ^13^C increased considerably in March (Fig. 8b). In contrast, δ^13^C fell with temperature and climate (Fig. 8c,d), δ^15^N also decreased during the same period (Fig. 9b) in response to an oscillation in temperature (Fig. 9c) and a rise in climate (Fig. 9d).

## Discussion

Despite a variety of methodological approaches for examining niche partitioning and assessing the complex trophic relationships, geographical trends between members of the same species, remain difficult to assess due to the high degree of similarity in isotopic ratio values. The present research attempts to address this not totally neglected topic^61,62^ and methodological gap^11^. Regardless of whether isotope data were derived from badger hair samples potentially including data from more than one badger, or samples where genotyping was used to account for individuals, linear regression analyses indicated that specific isotopic ratio values were associated with different land classes. Further, using soil isotope data as a baseline reduced error in the data. There was a distinction between animals living in upland and lowland land classes that can be noted. This was verified by the K-means cluster analyses, which consistently sorted the habitats into upland and lowland categories (Figs. 4 and 5) except for class C (Marginal Lowlands) which was spread along the full length of the regression lines, suggesting that additional factors are contributing to isotopic variation in this land class. In turn, this shows that badgers present in lowland regions have stable isotope values associated with a more predatory life-history, whilst those in uplands show evidence of a more omnivorous life-history, though there is likely variation between individuals (Figs. 2 and 3).

GAM models were used to account for isotopic variation with time of year, coupled with meteorological data, showing trends through time. Date of sample collection must be used in conjunction with meteorological data recorded from local weather stations, as regional data would not be able to account for the nuances seen across larger geo-spatial scales. The trend in meteorological and isotopic ratio data were detectable despite a limited period of collection, including a short time (late February and early March) when sampling was reduced. The analysis demonstrated that deducting the soil environmental baseline from isotopic ratios in animal tissue improves the signal in the data and potentially allows hidden biogeographical trends to be detected. For example, when the environmental baseline is used, it is evident that a badger δ^13^C value is dependent upon suitable meteorological conditions (Table 2). Additionally, the increase in the R^2^ values observed after the deduction of the environmental baseline in the regression models (Figs. 2a,b, 3a,b) can be interpreted as a reduction in the degree of variance and the underlying mean squares associated with each dataset (Table 1). This is important, given the innovations in isotopic research regarding trophic analyses^63^, the widespread use of isotopic techniques in macroscale studies, where both spatial and temporal heterogeneity are considered has remained a difficulty^21^.

Analyses of badger hair isotope ratios in Southern England revealed that badgers from the same sett/clan appear to develop independent foraging strategies to avoid intra-specific competition^37^. This creates isotopic variation between individuals within the same land class. These patterns are also apparent in Figs. 2 and 3, as some individual animals were from the same sett. This effect was evident in both the uncorrected and corrected datasets where the soil isotope baseline was deducted showing the strength of this signal within the data. The importance of isotope variation at the individual level, is strengthened further by the variation between different land classes and the much greater geospatial scale over which this population was studied, facilitating conclusions pertaining to the macro-scale. However, the degree of error was reduced in the corrected dataset and some groups changed position. Land classes B (Lakeland) and D (Central Lowland) exemplify this; in the uncorrected data, B ratios are higher than D (Fig. 2) but in the corrected data set D ratios are mostly higher than B (Fig. 3). This demonstrates the importance of the soil baseline. Without its use, it would be reasonable to conclude that badgers from Lakelands had a more protein rich diet than those in Central Lowlands (Fig. 2), but the corrected data shows that the opposite occurs and that the two groups have more similar diets than previously suggested (Fig. 3).

Other factors could contribute to the isotopic patterns observed. Northern Ireland like most of Western Europe, has a dense road network, but it is the connectedness of settlements and infrastructure in the local landscape not the underpinning land classification adjoining the road network which is a greater predictor of road traffic and road mortality in badgers^64^. In turn, the distinction between upland and lowland land classes, may be partially explained by roads creating ‘islands’ from which some badger populations do not stray. As roads are more prominent in lowland areas, particularly in the south-east of Northern Ireland and around Lough Neagh, associated with higher human population density. Badgers are also documented to prefer herbaceous fields and shrub lands, on podzol soils interspersed with rocky outcrops and an absence of cattle, as opposed to more intensively managed and desolate landscapes^65^. Furthermore, in Northern Ireland, badgers have higher population densities in semi-natural broadleaved forest and parkland habitats^36^. In the present study, badgers were recorded in all land-use types, except higher mountains (Fig. 1) and there was distinct patterning of isotopic ratios in all land-use types (Figs. 2, 3), except for land-use classes C (marginal lowland) and E (marginal uplands). This suggests that badgers in less intensively managed systems have an increased range in their isotopic ratios than those in other land-use classes and could be experiencing an anthropogenic constraint. Furthermore, lower productivity, scarcity of suitable sites for setts and adverse land management reduces badger density in uplands and marginal habitats^36,41^. Work on Amazonian River Otters (*Pteronura brasiliensis*) has shown that in resource poor environments, there are more incursions into neighbouring territories. Such excursions may also be necessary in marginal areas contributing to trends in Figs. 2 and 3, where there are notable crossovers between land-use types^66^. Territoriality is stricter in optimal habitats where highly complex olfactory communication, shared latrines, and gland secretions enforce territorial boundaries^67^. Thus, isotopic ratios of badgers have the potential to reveal aspects of social behaviour at a clan and population level, which may have a bearing on badger management in heterogeneous landscapes, and potential effects on badger-to-badger transmission of bovine Tb.

On a local scale, burrowing by badgers can affect local plant communities by turning over topsoil, affecting isotopic ratios on a local scale, but, on a macroscale, environmental and anthropogenic factors will contribute more substantially^68^. Their omnivorous diet, preference for protein-rich food, temporally varying nutritional requirements, and food availability, suggests that both the spatial and temporal component of isotopic variation should be assessed when characterising badger ecology^69,70^. The K-means cluster analyses in this report highlighted differences between lowland and upland populations, but the high number of clusters suggests there is still more variation than can be accounted for by differences at the level of land class (Figs. 4, 5). Environmental factors are the most likely source of this variation and have notable effects on badger physiology. The sampling period in the present study was short (February-April 2009) and reductions in δ^13^C and increases in δ^15^N (Figs. 6, 7, 8 and 9) suggest that the population was experiencing less than ideal conditions that restricted diet and impacted stable isotope values. This was perhaps unsurprising, given that 2009 experienced a detrimental snow event in late winter/early spring^59^. Subsequently, as Temperature (PC1) and Climate (PC2) are co-correlated with Date and there is a delay of at least two weeks between the weather event in February 2009 and the point of sample collection it can be inferred the effect on the badgers was negative. Furthermore, evidence for this period of heavy snowfall can be noted in the reduction of δ^15^N ratio and later foraging conditions improved into April (Figs. 8, 9). However, it is possible, had sampling been more consistent in this period, that the sampling error seen in the Figs. 6a, 7a, 8a and 9a may have been reduced.

Badger foraging is closely tied to climate, with rainfall, photoperiod and humidity having an effect in both drier and wetter years and foraging periods are longer in drier years^71^. Sett quality is also a crucial factor in badger thermoregulation as more insulated setts produce higher body weights in cubs in spring and may affect delayed implantation impacting on time of birth and cub development^72^. These stressors amongst others could all affect an animal’s isotopic ratios. It is important to consider that different patterns of isotopic fractionation can be seen across different tissues each referencing a different time window that may be of interest to a researcher^9^. Consequently, an isoscape as an environmental baseline could be widely utilised in research in terrestrial systems to study intraspecific population trends. Nevertheless, there should be discretion on the part of the researcher regarding confounding sources of isotope variation.

It is concluded that the method developed here using soil isoscapes as an environmental baseline is an acceptable approach to understanding species ecology on a macro-scale in omnivorous generalists such as badgers. Meteorological factors make it possible to track the stability of a population through time and offers a less invasive screening tool to measure shifts in a population throughout the year than more conventional, hands-on methods. This approach showed badgers in lowland habitat had diets higher in protein and the whole population were adversely affected by weather conditions. This technique will not track isotopic ratios through trophic levels, but it will allow a researcher to study resident populations over larger geospatial scales and assess how habitat and the environment affect broadly distributed populations. It is also a potential tool for monitoring populations, for example, in badgers it might detect changes in behaviour with potential ramifications for disease transmission.

## Supplementary Information

Supplementary Material 1-3.

Supplementary Material 4.

## Data Availability

The data that support the findings of this research are present in the Supplementary Material 4.
