# Author Correction: Soil δ^13^C and δ^15^N baselines clarify biogeographic heterogeneity in isotopic discrimination of European badgers (*Meles meles*)

**DOI:** 10.1038/s41598-022-09239-0

**Published:** 2022-03-24

**Authors:** Shay T. Mullineaux, Berit Kostka, Luc Rock, Neil Ogle, Nikki J. Marks, Rory Doherty, Chris Harrod, W. Ian Montgomery, D. Michael Scantlebury

**Affiliations:** 1 School of Biological Sciences, Queen’s University Belfast, 1‑33 Chlorine Gardens, Belfast, BT9 5AJ UK; 2 School of Natural and Built Environment, Queen’s University Belfast, David Keir Building, Stranmillis Road, Belfast, BT9 5AG UK; 3 Instituto de Ciencias Naturales Alexander Von Humboldt, Universidad de Antofagasta, Avenida Angamos 601, Antofagasta, Chile; 4 Universidad de Antofagasta Stable Isotope Facility (UASIF), Universidad de Antofagasta, Avenida Angamos 601, Antofagasta, Chile; 5 Shell Global Solutions International B.V., Amsterdam, The Netherlands

Correction to: *Scientific Reports* 10.1038/s41598-021-04011-2, published online 07 January 2022

The original version of this Article contained an error in the order of the Figures. Figures 7 and 8 were published as Figures 8 and 7. The Figure legends were correct at the time of publication.

The original Figures 7 and 8 and accompanying legends appear below.

**Figure 7:**
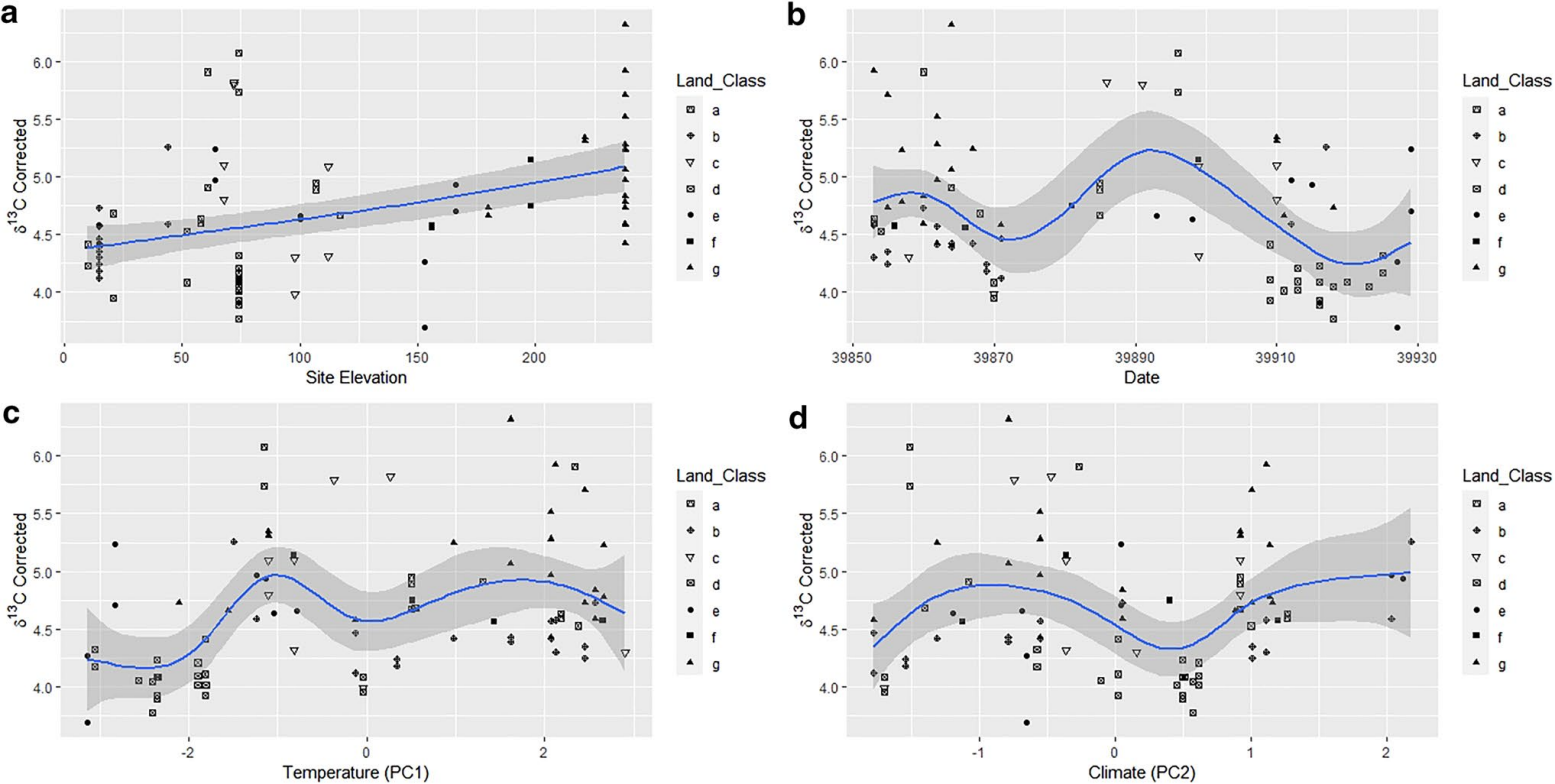
GAM factors smoothed for the hair dataset. Uncorrected δ^15^N versus (**a**) Site Elevation, (**b**) Date, (**c**) Temperature PC1, and (**d**) Climate PC2. Solid lines indicate the trend line, and the shaded area is the approximate 95% point-wise confidence interval.

**Figure 8:**
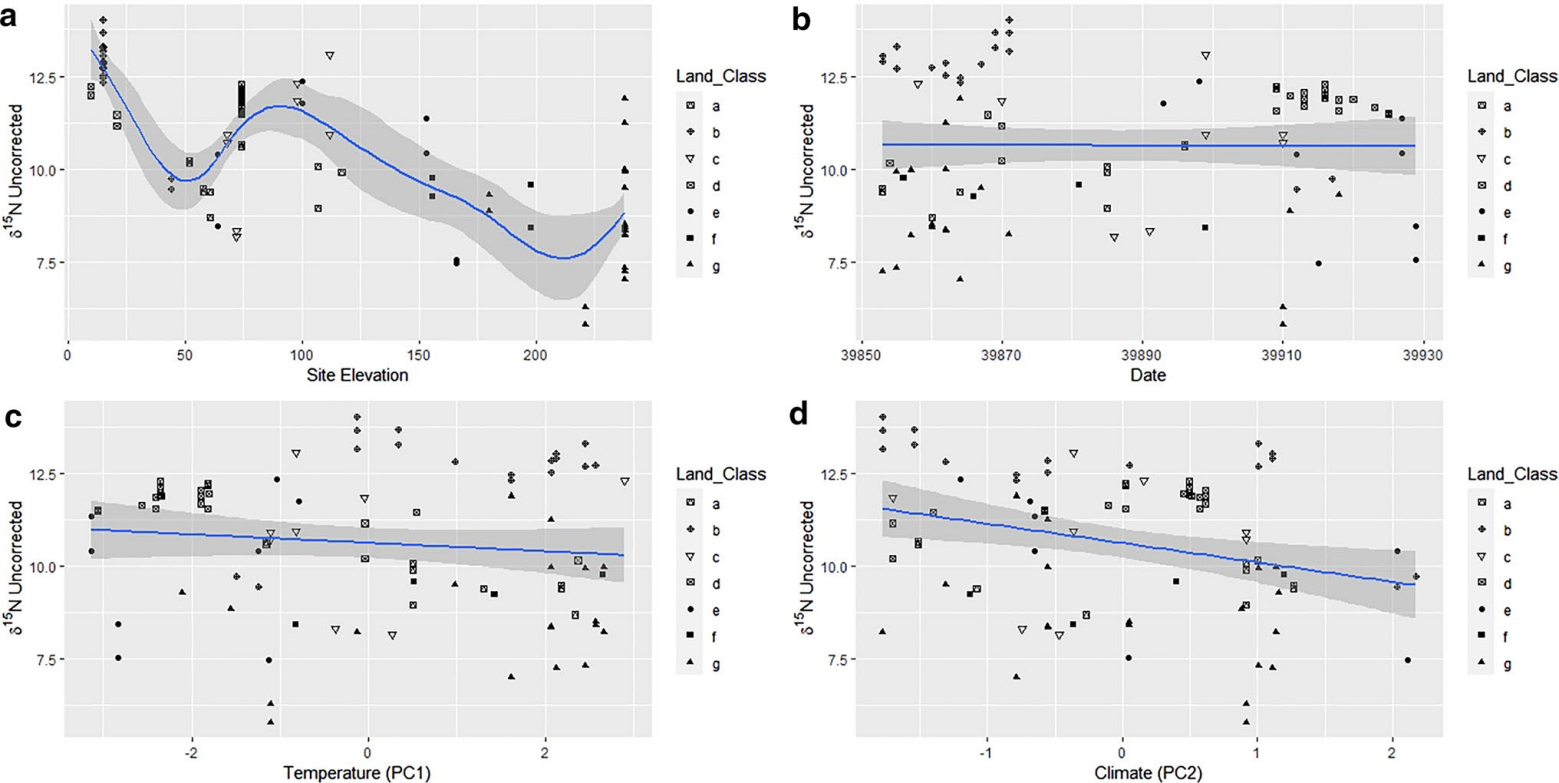
GAM factors smoothed for the hair dataset. Corrected δ^13^C versus (**a**) Site Elevation, (**b**) Date, (**c**) Temperature PC1 and, (**d**) Climate PC2. Solid lines indicate the trend line, and the shaded area is the approximate 95% point-wise confidence interval.

The original Article has been corrected.

