# Supplementary material for: Soil δ13C and δ15N baselines clarify biogeographic heterogeneity in isotopic discrimination of European badgers (Meles meles)
Source: Sci Rep. 2022 Jan 7;12:200. doi: 10.1038/s41598-021-04011-2 (PMC8741785; doi:10.1038/s41598-021-04011-2)
Supplement: Supplementary file 1 — Supplementary Material 1-3. [file 41598_2021_4011_MOESM1_ESM.docx]

Supplementary Material 1

Contained within are details on the distribution of the δ^13^C and δ^15^N baselines (Figures S1 and S2) and the land classes used in this study (Table S1). The baselines were created using ordinary kriging and black dots indicate locations of soil sampling. Additionally, further details on land classes can be seen in Table S1, detailing various environmental and anthropogenic factors.


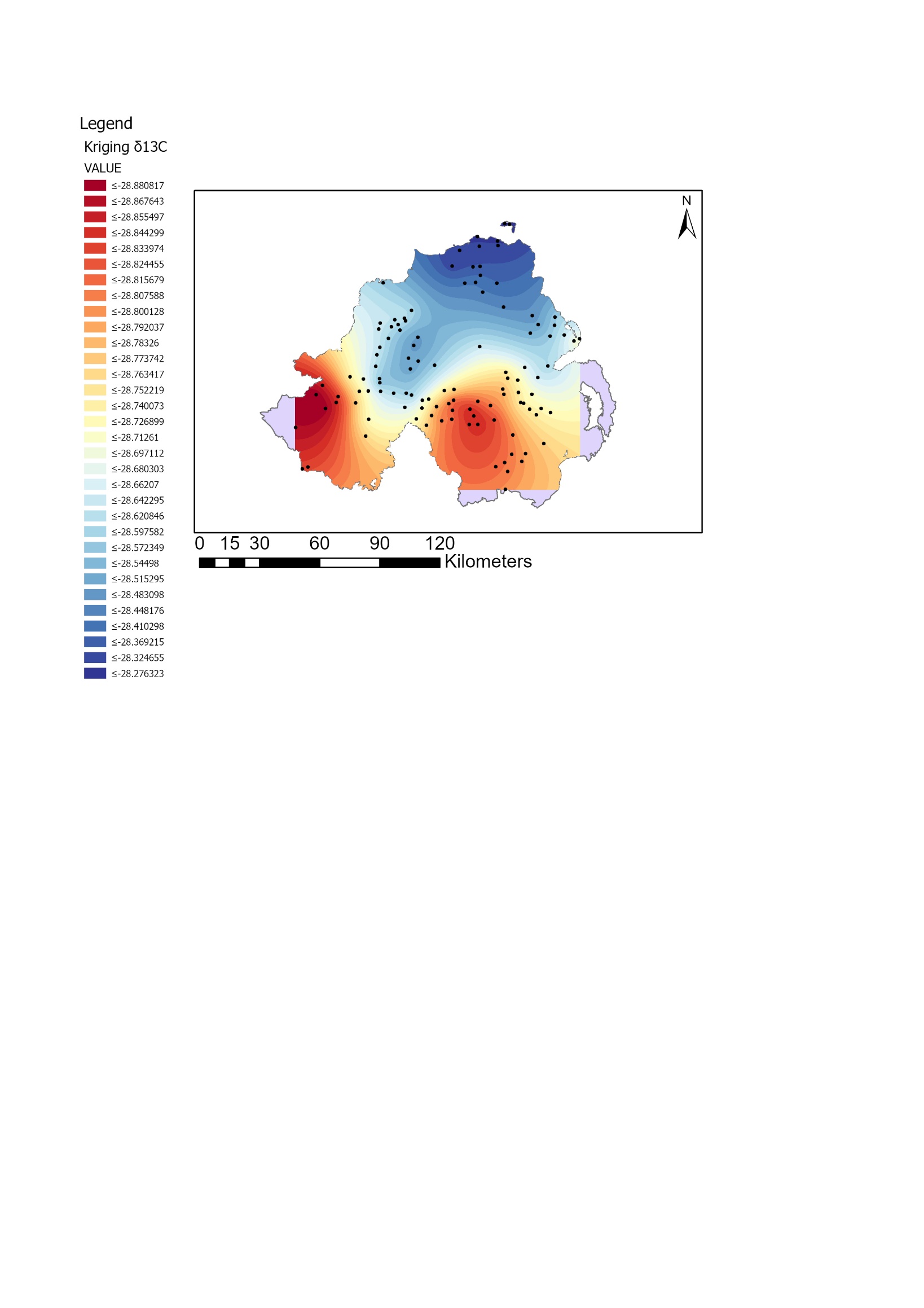


Figure S1: δ^13^C Isoscape of Northern Irish Soils


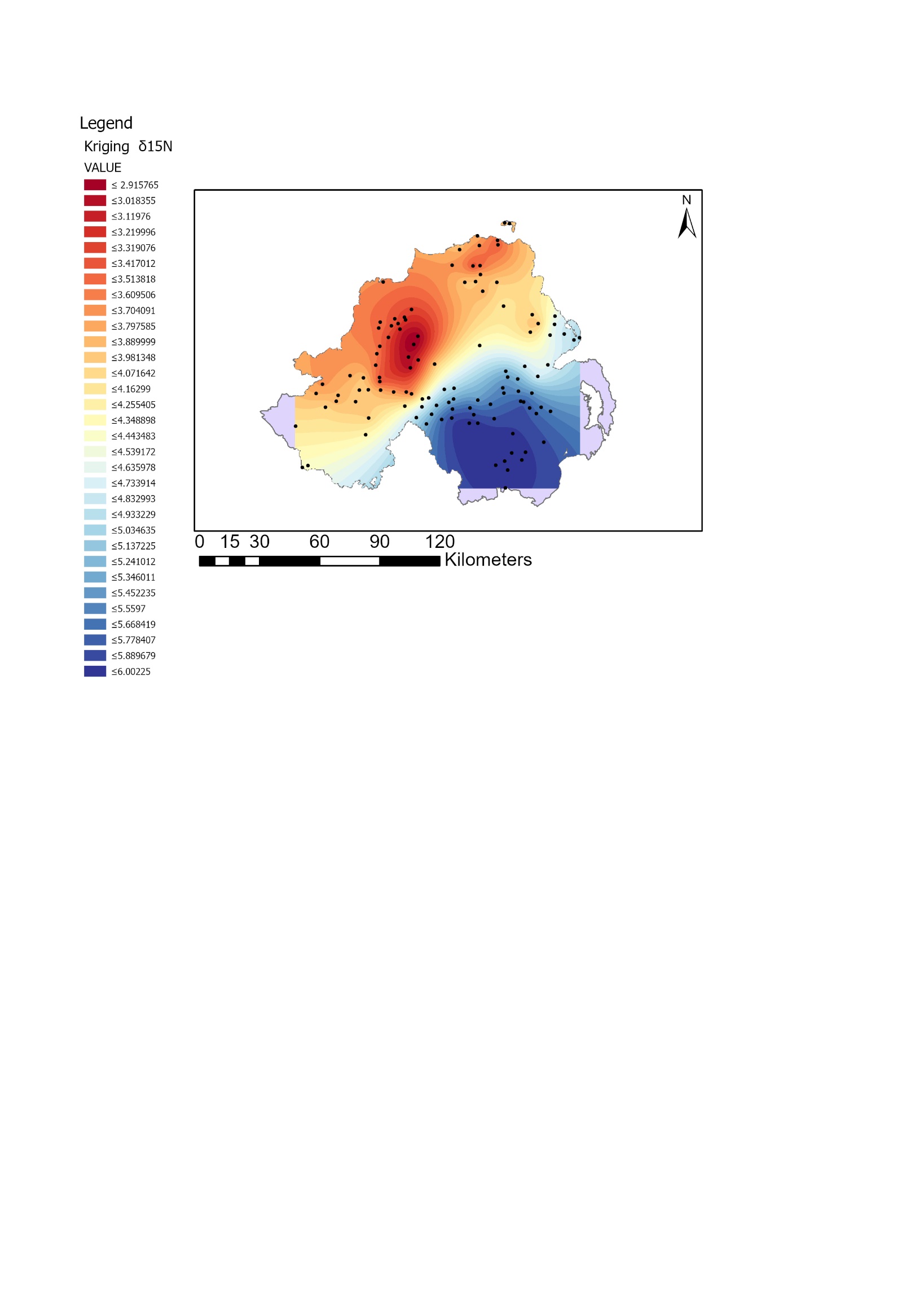


Figure S2: δ^15^N Isoscape of Northern Irish Soils.

Table S1: Details on all landscape types (Kostka, 2012; Murray *et al*., 1992).

| Land Class  (LC) | Elevation | Topography | Hydrology | Geology | Soils | Settlement | | Climate | | |
| --- | --- | --- | --- | --- | --- | --- | --- | --- | --- | --- |
|  |  |  |  |  |  | Road Network | Urbanisation | January temperature | July temperature | Sleet/Snow |
| A  Drumlin  Farmlands | 0—152 m | Flat and undulating with drumlin hills | Stream and River Network | Basic Igneous  Mixed limestone, shale and sandstone  Sandstone and conglomerates  Greywackes | Acidic brown earths  Brown/grey-brown podzols  Gleys | Developed, all road types | Intermediate | Moderate—high | Moderate—high | Low |
| B  Lakelands | 0—152 m | Flat and undulating | Stream and River Network | Mixed limestone, shale and sandstone  Sandstone and conglomerates  Pre-Cambrian limestones | Acidic brown earths  Brown/grey-brown podzols  Peaty podzols  Gleys | Developed, all road types | Moderate | Moderate—high | Low—moderate | Low—moderate |
| C  Marginal  Lowlands | 0—152m | Flat and undulating with drumlin hill bases | Stream and River Network | Basic igneous  Mixed limestone, shale and sandstone.  Sandstone and conglomerates | Acidic brown earths  Brown/grey-brown podzols  Gleys | Developed, all road types | Intermediate | Low—moderate | Low—moderate | Moderate—high |
| D  Central  Lowlands | 0—152 m | Flat and undulating | Stream and River Network | Basic igneous | Acidic brown earths  Gleys  Blanket peat | Developed, all road types | Intermediate | Low—moderate | Low—high | Moderate |
| E  Marginal  Uplands | 0—152 m | Slopes and drumlin hill-scape | Stream and River Network | Basic igneous  Mixed sandstone and conglomerates.  Pre-Cambrian limestones  Shales and mudstones  Shales and greywackes | Acidic brown earths  Brown/grey-brown podzols  Gleys | Developed, all road types | Intermediate | Moderate—high | Low—moderate | Low—moderate |
| F  Settled  Uplands | 153—244 m | Slopes and hill-scape | Stream Network | Basic Igneous  Granite and gneiss  Shales and greywackes  Sandstone and conglomerates | Acidic brown earths  Brown/grey-brown podzols  Gleys  Peaty podzols | Moderate, secondary, tertiary and minor roads | Moderate | Low—moderate | Moderate—high | Low—high |
| G  High  Uplands | 153—244 m | Slopes and elevated plateau | Stream Network | Basic igneous  Mixed limestone, shale and sandstone.  Sandstones and conglomerates  Schists  Chalk and associated strata.  Precambrian Limestones | Brown/grey-brown podzols  Gleys  Peaty gleys  Blanket peat | Moderate, secondary, tertiary and minor roads | Low | Moderate—high | Low—moderate | Moderate—high |
| H  Mountains | > 244 m | Slopes and mountain-scape | Stream Network | Basic igneous  Mixed limestone, shale and sandstone  Schists  Granite and Gneiss | Gleys  Peaty podzols  Peaty gleys  Blanket peat | Minor roads | Few | Moderate—high | Low—moderate | Low—high |

Supplementary Material 2

DNA extraction

There was a chance that hairs contained in one sample originated from different
individuals. Therefore, to obtain genotypes from one individual only, DNA was extracted
from single hair roots (using molecular facilities at Queen’s University Belfast, UK). This
technique has previously been shown to provide reliable genotypes (Sloane et al. 2000,
Frantz et al. 2004, Scheppers et al. 2007). A second DNA extract was prepared containing
all remaining hairs of one sample, as a ‘back-up’, should the single hair extract not contain DNA in an amplifiable amount (Scheppers et al. 2007). DNA was extracted using a
Chelex protocol (Chelex®-100, Bio-Rad, Hercules, CA; Walsh et al. 1991). Follicles were
cut off, transferred into 1.5 ml Eppendorf tubes containing 1.0 ml ddH_2_O and left to
incubate at room temperature for 30 minutes. After spinning each sample at 13 000 rpm
for three minutes the supernatant was removed. The samples were then washed again by
adding 1.0 ml of ddH_2_O and vortexing for 10-15 seconds after which the supernatant was
removed. Muddy samples were washed again until the supernatant remained clear. A
solution of 5% Chelex in low TE was then added to the samples and mixed well by hand
for two minutes. Samples were then incubated at 56ºC in a water bath for 45 minutes.
During the incubation all samples were mixed every 15 minutes, ensuring the follicle was
completely submersed in the Chelex solution. After incubation, the samples were mixed
again for two minutes. The samples were then boiled on a hotplate at 95ºC for eight
minutes, and again mixed directly afterwards. After spinning the samples at 13,000 rpm
for three minutes the supernatant containing the DNA extract was aliquotted into two
sterile 1.5 ml Eppendorf tubes and stored at -20 ºC.

Microsatellite PCR amplification

Twenty-two microsatellite loci derived from English badger populations
(Carpenter et al. 2003, Annavi et al. 2011) were tested for their applicability in obtaining
genetic profiles of badgers from the Northern Irish population. Testing procedures
involved adjustments of PCR reaction concentrations and PCR profiles and discarding
loci that failed to amplify or produce reliable allele peaks after genotyping. Therefore, in
order to maximise reliability and accuracy of genotypes, the number of loci used in this
study was reduced to seven, which was shown to be sufficient to identify individual
badgers (Frantz et al. 2004, Scheppers et al. 2007).

Individual genotypes were obtained by amplifying the microsatellite loci Mel-111,
Mel-116, Mel-117, Mel-246, Mel-451, Mel-554, and Mel-576 (Carpenter et al. 2003,
Annavi et al. 2011; Table 1), forward tailed with CACGACGTTGTAAAACGAC and
reverse tailed with GTGTCTT. Reactions were carried out in 10 µl reaction volumes,
containing 4 µl of DNA extract (1:5 dilution) and 1 pmol forward primer, 10 pmol
reverse primer, 10 pmol labelled M13 primer (6-FAM or HEX), 1x PCR reaction buffer,
200 µM each dNTP, 2.5 mM MgCl2 and 0.25 U Taq polymerase (GoTaq® Flexi DNA
Polymerase, Promega Corporation, Madison, USA). DNA amplification was carried out
using modified PCR profiles from Frantz et al. (2003) and Scheppers et al. (2007). All
PCRs started with a 5-minute denaturation period at 94°C. This was followed either by
45 touch-down cycles of 94°C for 45 seconds, annealing at 61 - 56°C for 1 minute and
72°C for 45 seconds, decreasing the annealing temperature by 1°C every cycle for six
cycles, and a final extension at 72°C for 5 minutes (Mel-111, Mel-116, Mel-117); or by
45 cycles of 94°C for 30 seconds, annealing at 55°C for 30 seconds and 72°C for 30
seconds, followed by a final extension at 72°C for 5 minutes (Mel-246, Mel-451, Mel-
554 and Mel-576). Microsatellite fragments were detected on an ABI 3730xl capillary
genotyping system (Applied Biosystems, Warrington, UK) and allele sizes scored using
LIZ-500 size-marker and GeneMarker® 1.8 genotyping software (SoftGenetics LLC®,
State College, PA, USA). Negative controls were included in PCR amplifications (Kostka, 2012).


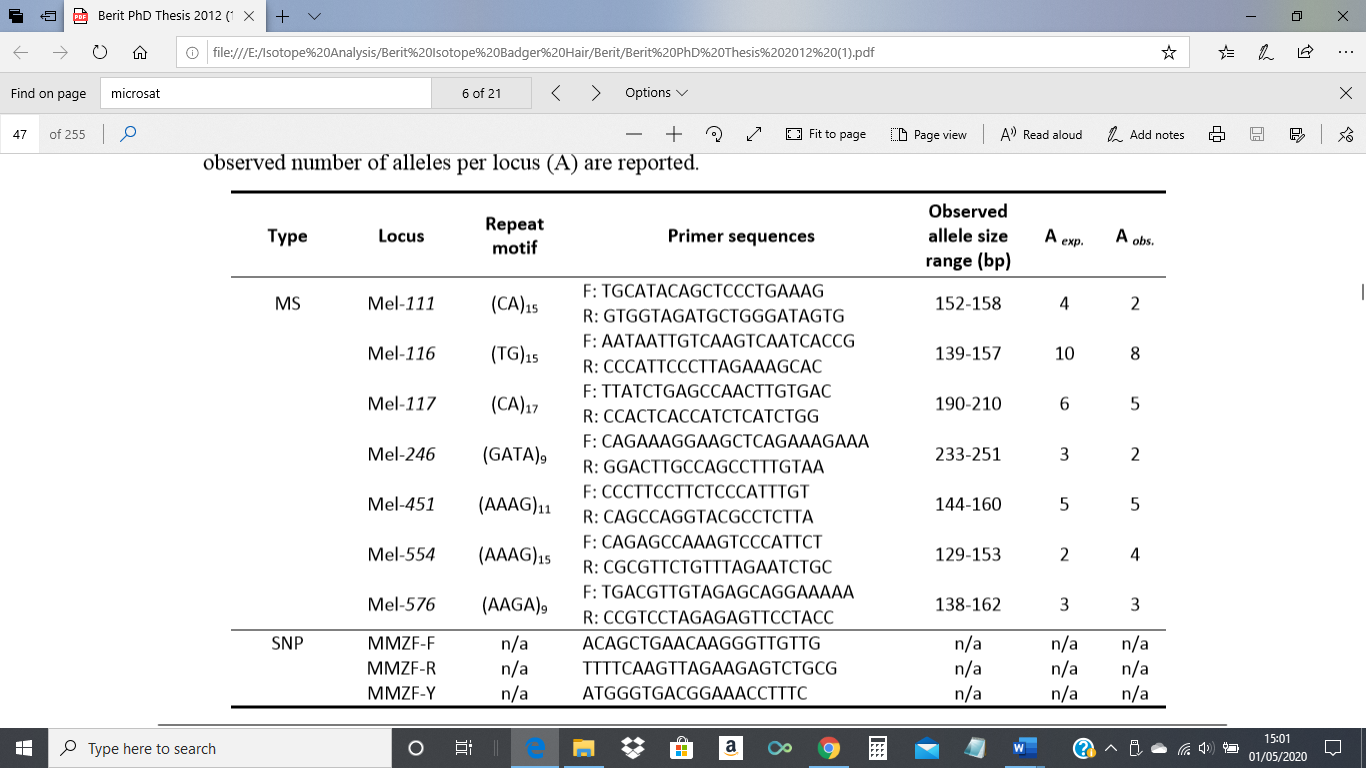
Table S1: Primer sequences of microsatellite (MS) and single nucleotide polymorphism (SNP) loci used to identify individual badgers. Repeat motif, observed size range (base pairs, bp) as well as expected and observed number of alleles per locus (A) are reported (Kostka, 2012).

Data quality control

Genetic material from non-invasively collected samples, especially hair, can be of low quantity and quality (Morin et al. 2001) and, hence, is prone to the occurrence of genotyping errors resulting in either too many or too few individuals detected by genotyping (Paetkau 2003). These can either appear in form of ‘allelic drop-out’, where one allele of a heterozygote is not detected (e.g. Gagneux et al. 1997, Taberlet & Luikart 1999) or by creating ‘false alleles’ during PCR amplification (Taberlet et al. 1996). Therefore, the risk of genotyping errors occurring in the current study was minimised by applying a very conservative approach to the scoring of alleles using the following methods: (1) microsatellite loci were amplified in a single-plex (one locus per PCR reaction) to avoid competitive amplification of loci during PCR that may result in false alleles or allelic drop-out (Taberlet & Luikart 1999); (2) tetra-nucleotide repeat loci were included to decrease the occurrence of stutter bands that can lead to false scoring of alleles (Taberlet & Luikart 1999, et al. 2001); (3) samples that did not amplify ZFY or ZFX were rejected due to insufficient DNA quantity for further analysis; (4) samples performing poorly during PCR or genotyping were repeatedly genotyped and rejected after a further two rounds of amplification if no complete genotype was obtained; (6) only genotypes of samples for which alleles were easily identifiable at each locus (e.g. strong peaks) were included in the final dataset. After completion of allele scoring, data were checked for null-alleles, allelic dropout and scoring errors using Micro-Checker 2.2.3 (Van Oosterhout et al. 2004) (Kostka, 2012).

References

Annavi, G., Dawson, D.A., Horsburgh, G.J., Greig, C., Dugdale, H.L., Newman, C., Macdonald, D.W. and Burke, T., (2011). Characterisation of twenty-one European badger (*Meles meles*) microsatellite loci facilitates the discrimination of second-order relatives. *Conservation Genetics Resources*, *3*(3), pp.515-518. doi.org/10.1007/s12686-011-9392-9

Carpenter, P.J., Dawson, D.A., Greig, C., Parham, A., Cheeseman, C.L. and Burke, T., (2003). Isolation of 39 polymorphic microsatellite loci and the development of a fluorescently labelled marker set for the Eurasian badger (*Meles meles*) (Carnivora: Mustelidae). *Molecular Ecology Notes*, *3*(4), 610-615. [https://](https://doi.org/10.1146/annurev-ecolsys-102209-144636)[10.1046/j.1471-8286.2003.00529.x](https://doi.org/10.1046/j.1471-8286.2003.00529.x)

Frantz, A.C., Schaul, M., Pope, L.C., Fack, F., Schley, L., Muller, C.P. and Roper, T.J., (2004). Estimating population size by genotyping remotely plucked hair: the Eurasian badger. *Journal of Applied Ecology*, *41*(5), 985-995. <https://doi.org/10.1111/j.0021-8901.2004.00951.x>

Gagneux, P., Boesch, C., & Woodruff, D. S. (1997). Microsatellite scoring errors associated with noninvasive genotyping based on nuclear DNA amplified from shed hair. *Molecular ecology*, *6*(9), 861-868. <https://doi.org/10.1111/j.1365-294X.1997.tb00140.x>

Kostka, B.I., (2012). *Landscape ecology, diet composition and energetics of the Eurasian badger (Meles meles).* Unpublished PhD thesis, Queen's University Belfast. https://ethos.bl.uk/OrderDetails.do?uin=uk.bl.ethos.579755

Paetkau, D., (2003). An empirical exploration of data quality in DNA‐based population inventories. *Molecular ecology*, *12*(6), 1375-1387. <https://doi.org/10.1046/j.1365-294X.2003.01820.x>

Scheppers, T.L., Roper, T.J., Frantz, A.C., Schaul, M., Engel, E., Breyne, P. and Schley, L., (2007). Estimating social group size of Eurasian badgers *Meles meles* by genotyping remotely plucked single hairs. *Wildlife Biology*, *13*(2), 195-207. https://doi.org/10.2981/0909-6396(2007)13[195:ESGSOE]2.0.CO;2

Sloane, M.A., Sunnucks, P., Alpers, D., Beheregaray, L.B. and Taylor, A.C., (2000). Highly reliable genetic identification of individual northern hairy‐nosed wombats from single remotely collected hairs: a feasible censusing method. *Molecular Ecology*, *9*(9), 1233-1240. <https://doi.org/10.1046/j.1365-294x.2000.00993.x>

Taberlet, P., & Luikart, G. (1999). Non-invasive genetic sampling and individual identification. *Biological journal of the linnean society*, *68*(1-2), 41-55. <https://doi.org/10.1111/j.1095-8312.1999.tb01157.x>

Taberlet, P., Griffin, S., Goossens, B., Questiau, S., Manceau, V., Escaravage, N., ... & Bouvet, J. (1996). Reliable genotyping of samples with very low DNA quantities using PCR. *Nucleic acids research*, *24*(16), 3189-3194. <https://doi.org/10.1093/nar/24.16.3189>

Van Oosterhout, C., Hutchinson, W. F., Wills, D. P., & Shipley, P. (2004). MICRO‐CHECKER: software for identifying and correcting genotyping errors in microsatellite data. *Molecular Ecology Notes*, *4*(3), 535-538. <https://doi.org/10.1111/j.1471-8286.2004.00684.x>

Walsh, P.S., Metzger, D.A. and Higuchi, R., (1991). Chelex 100 as a medium for simple extraction of DNA for PCR-based typing from forensic material. *Biotechniques*, *10*(4), 506-513. 10(4):506-513

Supplementary Material 3


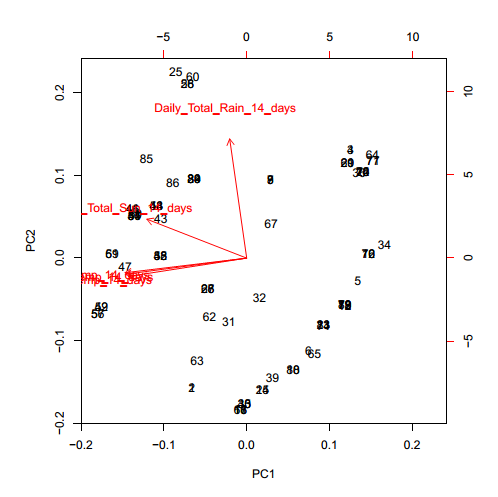


Figure S1: PCA Biplot for meteorological data, averaged over 14 days before hair extraction, factors include: mean daily temperature (⁰C), maximum daily temperature (⁰C), minimum daily temperature (⁰C), daily rainfall (mm) and daily sunshine hours (hrs). The three temperature variables are highly congruent with almost all variance accounted by PC1, daily rainfall (mm) is the most distinct and is mostly accounted for by PC2 and daily sunshine hours (hrs) is split between both PC axes.


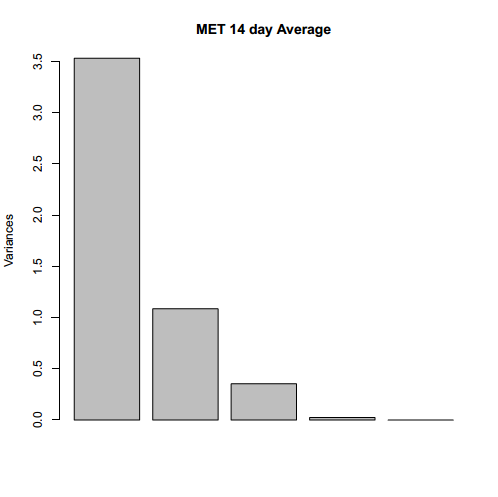


Figure S2: PCA scree plot for meteorological data averaged over 14 days before hair extraction, most variance explained by PCs one and two. The vast majority of variance is accounted for PC1 (71% of variance) temperature and PC2 (22% of variance) climate.
